# Evaluating the Technology Readiness of a Ribbon-Blade Wind Turbine Using NASA’s TRL Method

**DOI:** 10.3390/ma14247709

**Published:** 2021-12-13

**Authors:** Robert Kasner, Patrycja Bałdowska-Witos

**Affiliations:** Department of Machines and Technical Systems, Faculty of Mechanical Engineering, Bydgoszcz University of Science and Technology, Al. Prof. S. Kaliskiego 7, 85-796 Bydgoszcz, Poland; robert.kasner@pbs.edu.pl

**Keywords:** innovative wind ribbon turbine, verification of the method of assessing the level of innovation readiness

## Abstract

The aim of this article was to complete a methodologically original study and evaluation of the technological readiness of an innovative ribbon-blade wind turbine in accordance with NASA’s TRL method. The structural form of the wind turbine unit analyzed herein, featuring a new ribbon turbine design, is distinguished by its safe durability. The circumferential speeds of the points on the turbine circumference were technologically verified positively and have a significant impact on the evaluation indicators of the conversion process, these being efficiency, unit energy consumption, and the quality of the power and energy of the wind power plant. The use of a new turbine design in the wind turbine analyzed herein, a working ribbon unit, resulted in a technological increase in efficiency from 13% to 32% and a reduction in unit internal energy consumption from 18% to 36% compared to the traditional wind turbine design. The TRL NASA-based evaluation herein, which consists of modern computer-aided engineering procedures (CAE standard) as well as IT instrumentation, and which includes nine degrees of technological readiness of an innovative ribbon windmill, falls in line with the standards for smart development based on knowledge and innovation (EU 2020 Strategy).

## 1. Introduction

Researching, developing, and assessing the technological readiness of an innovative ribbon turbine using NASA’s TRL method falls in line with the realization of intelligent development goals laid out in the “Europe 2020 Strategy for Development” [1,2,3,4].

Bioenergy, derived from environmental sources, is the second-largest natural energy resource utilized in the world [5,6,7]. Essential parameters of this process, such as changes in the quality of power and energy, must be improved in order to meet the needs of consumers [8,9,10,11,12]. Before introducing the innovative turbine to the market, it is necessary to assess its technological readiness and maturity in the context of both commercialization and the benefits from its use [2,3,13]. This assessment encompasses the current and factual state of work relating to the turbine’s progress as well as to development prospects, the amount of money needed to invest in the product, and technological risk. Technology Readiness Levels constitute a method tried and tested in appropriate conditions necessary to assess the development levels of new ideas, structures, processes, and projects of a technological-research nature. The innovative wind turbine examined herein was evaluated as a product prototype in simulated conditions similar to actual ones, this being one of the most important stages of the TRL method [1,14,15,16,17].

Wind energy is the cheapest renewable energy source in the world. The production of energy from renewable sources in Poland started to develop on a larger scale after 2005. During the last 10 years of wind energy development, the power utilization ratio in Poland has remained within the range of 20–26 percent. The high value of the degree of utilization of the installed capacity proves that in Poland, already in the first stage of the sector’s development, modern wind turbines with high efficiency of power use and low failure rate were used. In 2011, when wind conditions improved significantly, production increased by 20%. compared to the same year 2008, the value of the coefficient also increased. The power utilization factor depends primarily on factors such as wind conditions in the location of individual wind farms and the quality and efficiency of the installed turbine. The efficiency of windmills has also increased. Modern, well-located onshore wind turbines have power utilization rates above 25%. (in the best locations over 35%). Offshore wind farms have much higher efficiency—over 40%, and those in recent years, using the latest technologies, even 50%. The main benefits are the almost continuous wind, low production costs and long-term use. Increased reliability in electricity production and low environmental impact promise a positive future.

The aim of this work was to complete a methodically original investigation and evaluation of the technological readiness of an innovative ribbon windmill in accordance with NASA’s TRL method. To uphold an appropriate level of originality, it was necessary to complete an analysis of the current state of knowledge and technology in the field of innovation strategy and technological development and to assess the degree of technological readiness specific to the construction and operation of innovative machinery.

## 2. Solving the Task

To achieve the objectives described above the following question was formulated: is it possible to analyze, test, and assess the technological readiness of an innovative ribbon wind-blade turbine by applying NASA’s original TRL method?

Specifically, the practical assessment tasks can be reduced to answering two questions:

1. Will the use of an innovative ribbon-blade wind turbine contribute to efficiency gains and to a reduction in the energy used in the innovative process?
2. Will the use of said ribbon-blade turbine improve the effectiveness and quality of the product: power and electricity?

When approaching innovation it is possible to distinguish three original integrated methodological strategies of action which can be labeled as the Japanese, European and American approaches [8,9]. The Japanese strategy includes metatheory, discovery, enlightenment (i.e., the configuration determining the emergence of a new, more perfect level of machinery and equipment, where this configuration arises as a result of the influence of an undefined area of side factors that do not fall within the scope of phenomena that can be presented on the basis of the principles of the old level) and, lastly, practical usefulness (assuming, for example, implementation, the elimination of transmission losses, fuel savings, heat, electricity, etc.) and novelty diffusion. The European innovation strategy includes theory, a mathematical model, a new idea, new construction of the technical means and/or new parameters relating to the technological process. The American innovation strategy consists of analyzing the rise of a new need, and, accordingly, aims to satisfy that said need. This strategy presupposes that technological possibilities exist to meet this new need and that the new idea, means, or process are what primarily fuel prosperity [18,19]. Innovation strategies fit into the numerous methodological pro-implementation proceedings of the Bydgoszcz University of Science and Technology [20,21,22,23,24]. Such research, in tandem with optimization and efficiency measures (understood as design-conceptualization that meets efficiency criteria), leads to the creation of numerous solutions in machines, in this case, wind turbines. Energy conversion-related aims, phenomena simulators, and research instrumentation are essential in the implementation process of innovative wind turbines, especially insofar as the design features and process parameters of the unit are concerned [25,26,27,28,29,30,31,32,33,34,35].

## 3. The Innovation Readiness Method

Various models for assessing technology are in use. Those which are the most widespread include the TRL model (Technology Readiness Levels) [1,2,3,21].

The TRL method considers assessing the maturity of innovative technologies an important element in the decision-making process regarding commercialization. This assessment covers multiple areas: the level of work on the development of new technology, the potential for further development, and the level of financial resources needing to be invested, including investment risk. This is the Technology Readiness Assessment (TRA), which delivers information to persons responsible for decision-making. The TRL method is meant to be a unified metric utilized to analyze the progress of work on technologies as well as their readiness for implementation on the market. The TRL method does not concern the full commercialization process; it does not, among other things, claim whether there is demand for a given product or technology. Technology readiness levels were defined in a nine-degree scale presented in Table 1 [1,2,3,21,34].

## 4. Results, Discussions and Analysis of a Technology Readiness Assessment

The testing and assessment of the technological readiness levels of an innovative ribbon wind turbine in accordance with NASA’s TRL method were conducted while adhering to the principles of electrical power engineering and mechanical engineering specific to the construction and operation of innovative machinery. The results are as follows, organized according to particular TRL levels.

### 4.1. TRL 1: Identifying Basic Operating Principles

**Figure 1:**
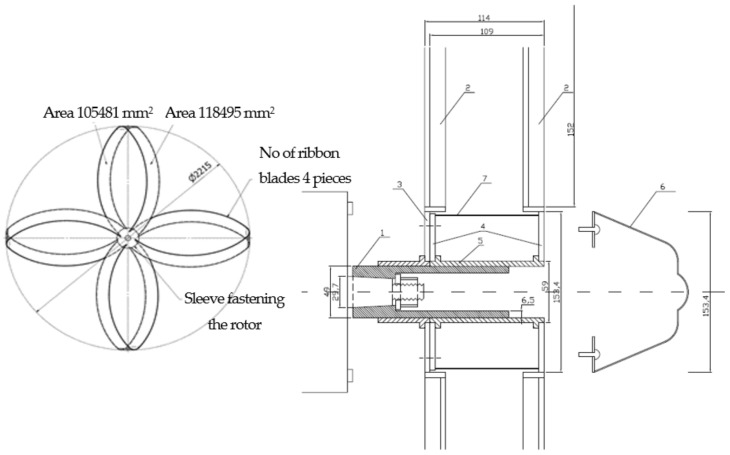
Construction of the rotor: geometrical shape with dimensions: 1. Main sleeve, 2. Fastening the web, 3. Main sleeve flange, 4. Flange movable sleeve, 5. Movable sleeve, 6. Front cover, and 7. Side cover. Source: own materials.

The new idea, design, and movement parameters of the wind turbine: the engineering essence of the conceptual solution of the ribbon-blade wind turbine (Figure 1) lies in the horizontally rotating rotor with radially arranged ribbon blades forming a figure-eight that are cross-mounted to form four loops of rotor blades offset from each other by 90°. The functional system that processes and converts energy—comprising a hub, gearbox, generator, tower and servo—form the wind turbine for electricity production.

A ribbon is a compact, two-dimensional topological manifold existing in three-dimensional space and can be obtained by welding together, end to end, a strip of structural material such as steel so that the double loop in the shape of a figure eight forms two sides, an inner one and an outer one. A ribbon blade formed in this way grants a spatial shaping possibility such that in the center of the longitudinal symmetry and in the middle of its circumference a point of attachment is formed in the axis of the turbine hub; moreover, the space for control, regulation and functional (working) compensation (coupled to the wind speed) is possible by changing this space’s geometric form via smooth adjustment of the angle of the blades forming the individual loops in the hub (Figure 1).

The kinematic system of the device consists of two bearing shafts (that of the slow-speed turbine and that of the fast-speed generator) connected by the accelerating gearbox. At the entrance of this system is the rotor with the hub and ribbon blades cross-mounted, while the electric generator is mounted at the exit of the fast-speed bearing shaft. The body of the device is set rotationally (the servo) on a steel tower with an angle of rotation corresponding to the most favorable local wind, terrain and operational conditions. The stabilization of the wind turbine is provided by the foundation and the strength of the tubular (conical), truss tower and by anchoring in the ground.

The construction of the wind turbine is based on a small number of components. The slow-speed rotor and the body (nacelle) of the housing of the fast-rotating devices inside (gearbox, shaft, and generator) prevent the destruction of living organisms such as birds, bats, insects, among others. The construction of the device may additionally be equipped with a grating that protects the rotor. The arrangement and shape of the ribbon blades (cross-mounted in the shape of a figure of eight) generates high torque, and the multiple radially mounted ribbon rotor blades with a smoothly varying angle of attack minimize rotor drag. The ribbon shape of the working surface of the blades additionally strengthens the lift effect and reduces frontal resistance thereby contributing to increases in the machine’s overall efficiency. The rotor of the wind turbine, thanks to its ribbon-shaped, radially set blades, turns the kinetic energy stream of the translational motion of the wind (air) into mechanical kinetic energy of rotational motion. Rotational motion is made use of to drive an electricity generator via the kinematic system with the accelerating gearbox. The remainder of this article will focus exclusively on the rotor itself. This results from the fact that only the rotor will be subject to durability and efficiency analysis. The rotor’s construction, geometric features, and geometric form are shown in Figure 1, in addition to relevant dimensions. In order to obtain a product of higher quality (with respect to power and energy), a multi-level, multi-stage conversion of energy (power) may be used. Rotors coupled rotationally can work together on the same plane (Table 2) [26,27,28,29,30,35].

### 4.2. TRL 2: Formulating the Concept of the Solution

Kinematics and dynamics of a new concept of wind turbine: The structure of a wind turbine allows for its operation in winds directed toward the rotor from various angles (Table 3). However, durability and efficiency analysis will here be conducted only with respect to wind along the rotational axis of the rotor. As seen in Figure 1, surface projections in the windward direction were calculated and served to define the forces applied to them.

Assessment result of TRL level 2 is presented in Table 4.

### 4.3. TRL 3: Validating the Proof-of-Concept Experimentally

**Figure 2:**
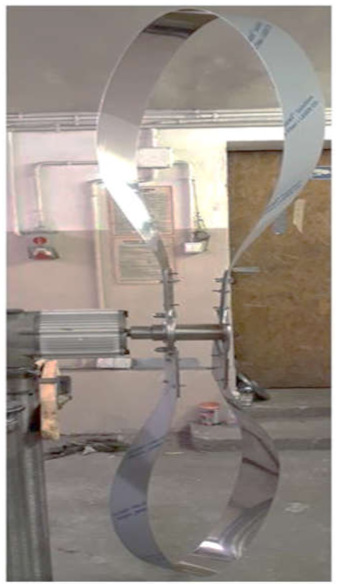
The manufacturing model of the rotor with ribbon blades. Source: the authors’ own materials.

**Figure 3:**
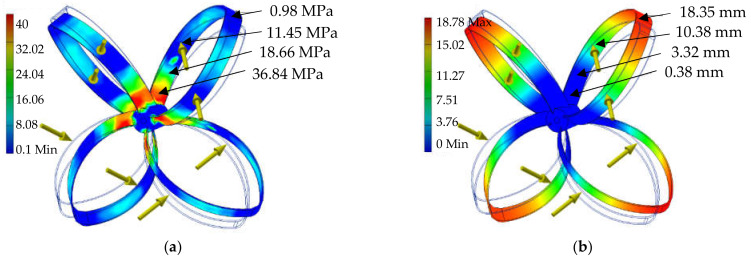
(**a**) von Mises stresses of the rotor; (**b**) displacement of the loaded rotor planes. Source: the authors’ own materials.

Testing the proof-of-concept involved determining the assessment indicators relating to product quality and energy process efficiency. Model new concept of wind turbine shown in Figure 2. A simulation of the finite element method type was conducted using the student version of Autodesk Inventor 2016. Figure 3 presents the points and directions of the forces applied on the rotor surfaces during the simulation.

The visible forces used for simulation purposes were taken as P_OB_ (Circumferential Force). There are two sizes in the calculations, and they are dependent on the area as shown in Figure 1. The whole structure was immobilized in the axis of rotation and the load arising at this point is transferred entirely to the turbine’s foundation.

Test results: the construction quality in a permissible area, the product quality (speed, resultant force, circumferential force), process efficiency, product safety, and process safety were positive and better than in the case of standard wind turbines of the same class with regard to the power-demand for operational motion (Table 5).

### 4.4. TRL 4: Validating the Technology in Laboratory Conditions; TRL 5: Validating the Technology in Simulated Operational Conditions

Validating the technology in laboratory conditions, in simulated operational conditions: the calculated forces for a wind speed of 47 m·s^−1^ yield a circumferential force of 237 N and a unit force of 266 N. A wind speed at this level was assumed because it is the maximum accepted value which should translate into sufficient durability during use.

The rotor ribbons were designed from steel AISI 304. Their mechanical properties are shown in Table 6.

The rotor ribbon was designed in the form of a 110 mm × 1 mm × 2300 mm. A model designed in this way was tested in a simulation which yielded the following results.

**Figure 4:**
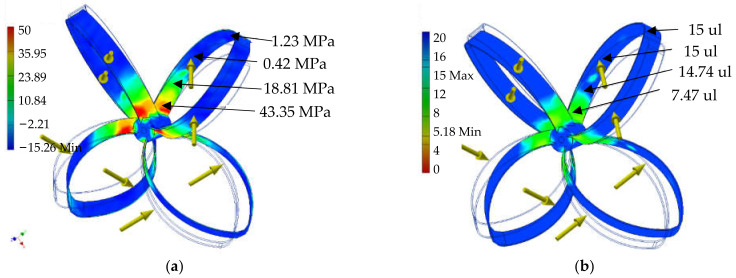
(**a**) distribution of first principal stresses on the ribbon surfaces; (**b**) distribution of the safety coefficient on the ribbon surfaces. Source: the authors’ own materials (based on M.M. Szarek).

From the results obtained the conclusion can be made that the sections used in the construction are able to carry the theoretical loads that occur. Assuming a conventional yield strength of 210 MPa with a safety coefficient xe = 4, we can determine that the stresses permissible in the construction being tested can reach 60 MPa. In Figure 3a the von Mises stresses reach a maximum value of 40 MPa while in Figure 4a the first principal stresses show a maximum stress of 44 MPa. These results, in combination with the distribution of the safety factor in Figure 4b confirm the validity of the design used.

Figure 3b presents the displacement that occurs during the theoretical operation of the rotor. This can be used when designing the remaining elements of the wind turbine. By predicting possible rotor deformations, it is possible to predict the distances that must be designed for the correct, collision-free operation of the entire system (Table 7). After successful completion of the laboratory verification stage, the adopted technology concept was confirmed. The positively verified components of the technology were integrated, reaching the TRL level 4. At this stage, the high quality of the verified components was obtained.

At level 5, the components of the ribbon wind farm were verified in an environment similar to the real one. The basic components of the technology are integrated with the real supporting elements. The technology can be tested under simulated laboratory conditions. At level 5, it was necessary to verify whether the ribbon rotor working unit was designed correctly and whether the results of the first simulation tests confirmed the correctness of the given mechanical and design properties.

**Figure 5:**
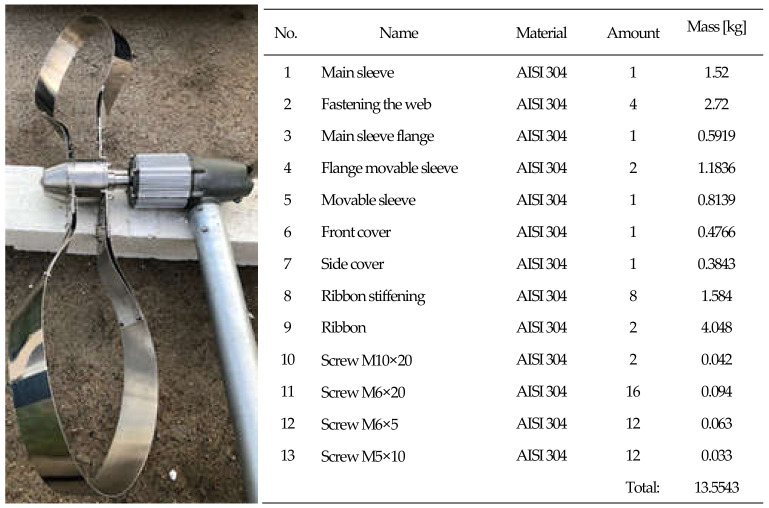
The real object with a list of components and materials for LCA.

Life Cycle Impact Assessment was performed using SimaPro 8.4 software (PRé Sustainability, LE Amersfoort, The Netherlands) with Ecoinvent 3.4 database. The cut-off level adopted for the research was 0.5% [36]. The environmental analysis of the life cycle of wind power plants construction elements was possible thanks to the use of two methods: IPCC 2013 (Intergovernmental Panel on Climate Change—carbon footprint) and CML 2 (Center of Environmental Science of Leiden University). The methods are used to present the impact of products and technologies on greenhouse gas emissions [37]. The functional unit adopted wind turbine divided into construction elements. The results of the collected and ordered input data are shown in Figure 5.

**Figure 6:**
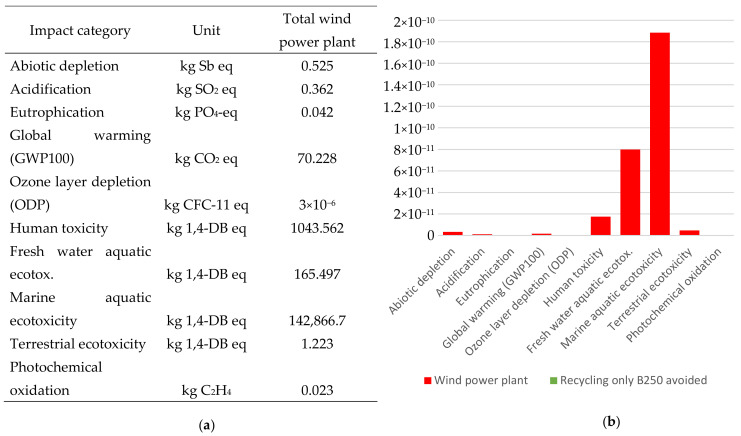
(**a**) Validation of the ribbon rotor manufacturing technology; (**b**) Results of the analysis and evaluation of the manufacture and disposal of rotor materials using the LCA procedure.

The assessment of the environmental impact of wind power plant construction elements using the IPCC 2013 method is shown in Figure 6. Data of indicators global warming (kg CO_2_ eq) and renewable wind energy (MJ) are presented in Table 8. Based on the analysis, it was found that the overall environmental indicator Global Warming Potential technology wind power plant amounts to 70 kg of CO_2_ eq/1p. The indicated parameter is an element of characterization and it has been presented for a hundred-year period. The lowest level of potential environmental damage was characterized by Ozone Depletion Potential, which for the entire wind power plant was 3×10^−6^ kg CFC-11 eq. It influences the depletion of the ozone layer, which contributes to an increased level of ultraviolet radiation amount of negatively affecting the health of humans and animals. In turn, the highest potential level of environmental damage was found in two categories: marine aquatic ecotoxicity and human toxicity (appropriately 142,866.7 kg 1,4-DB eq oraz 1043.562 kg 1,4-DB eq). The main problem in this category is the toxic substances in the human environment. Marine aquatic ecotoxicity relates to the effects of toxic substances on marine aquatic ecosystems. Any substance emitted to air, water or soil is included.

Figure 6 presents the data for the analysis of the technology in the conditions of producing a wind power plant.

### 4.5. TRL 6: Production and Demonstration of a Prototype in a Simulated Environment Similar to the Target One; TRL 7: Prototype Demonstration in the Target Environment

Prototype demonstration in a simulated environment similar to the target one and Prototype demonstration in the target environment: Research and development results concerning the ribbon-bladed wind turbine with a horizontal axis of rotation designed to convert the energy of air mass movement into torque on the axis of the main turbine shaft.

On the sixth level, the prototype was demonstrated in conditions similar to real life. Under the adopted operating conditions, the safety parameters of the ribbon operation of the wind turbine were assessed, including the verification of strength and peripheral velocities of points on the circumference of the turbine, which were positively verified. On the seventh level, the prototype of the ribbon wind power plant was demonstrated in near-real conditions. As part of level 7, the characteristics of the generated power as a function of the rotational speed of the rotor were removed.

The primary objective herein was to develop and produce a modernized rotor of a ribbon-blade wind turbine. Design to and set up research stations with an installation verifying the environmental parameters of operation so as to make it possible to set wind turbine parameters for theoretical and practical efficiency analysis and to assess the ribbon-blade wind power plant design. The research station project entailed creating three wind turbines to verify the performance of various types of rotors in actual conditions.

**Figure 7:**
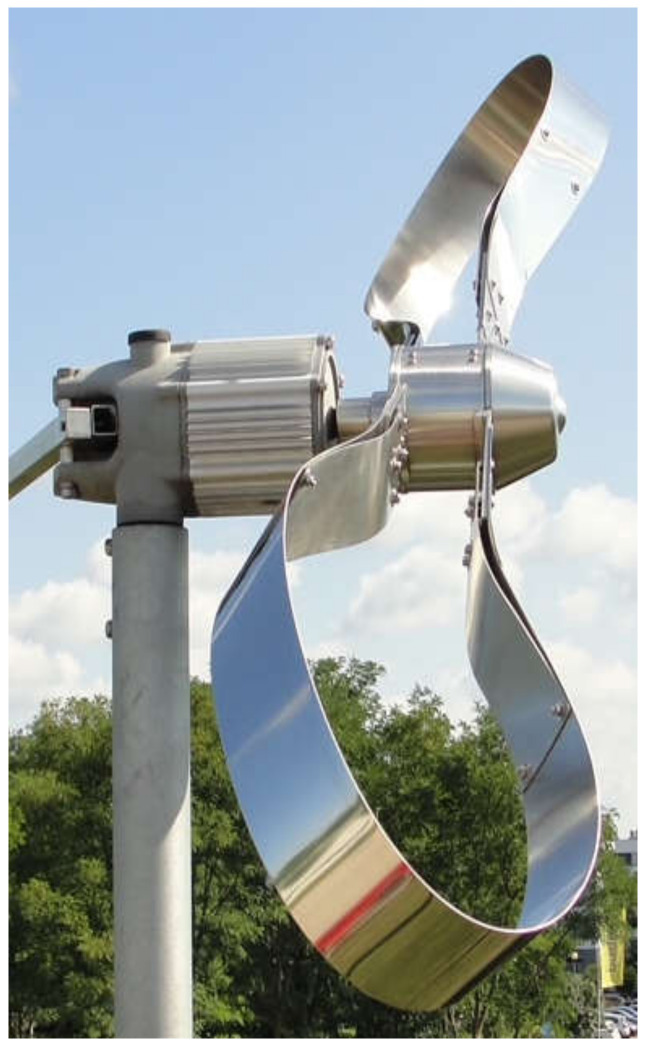
Wind power plant of a modernized ribbon-blade wind turbine under real conditions. Source: the authors’ own materials (based on R. Kasner).

**Figure 8:**
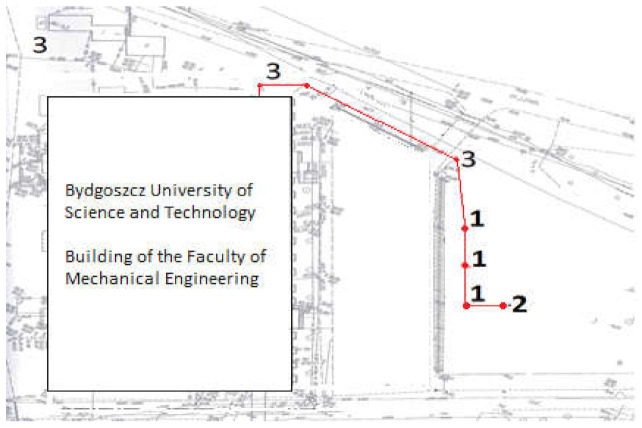
Geodetic inventory of wind turbine test stations: (**1**) wind power research positions; (**2**) environmental parameters measurement station; (**3**) telecommunication sewage system for monitoring. Source: the authors’ own materials (based on R. Kasner).

**Figure 9:**
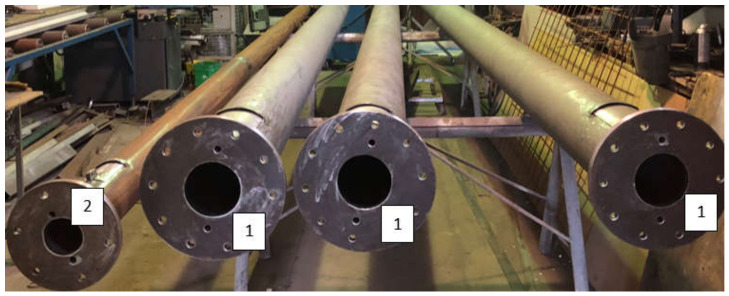
The completed tube construction of the wind turbine tower (**1**) and wind measurement stations (**2**). Source: the authors’ own materials (based on R. Kasner).

**Figure 10:**
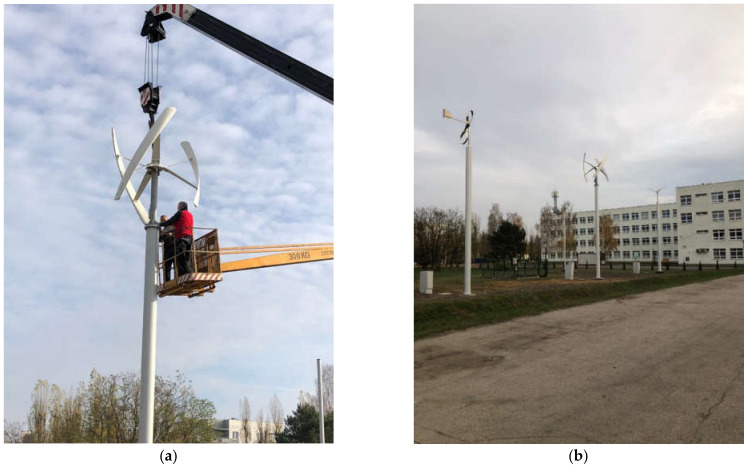
Assembly of the wind turbines at the testing station (**a**) the testing station of the wind turbines (**b**). Source: the authors’ own materials (based on R. Kasner).

**Figure 11:**
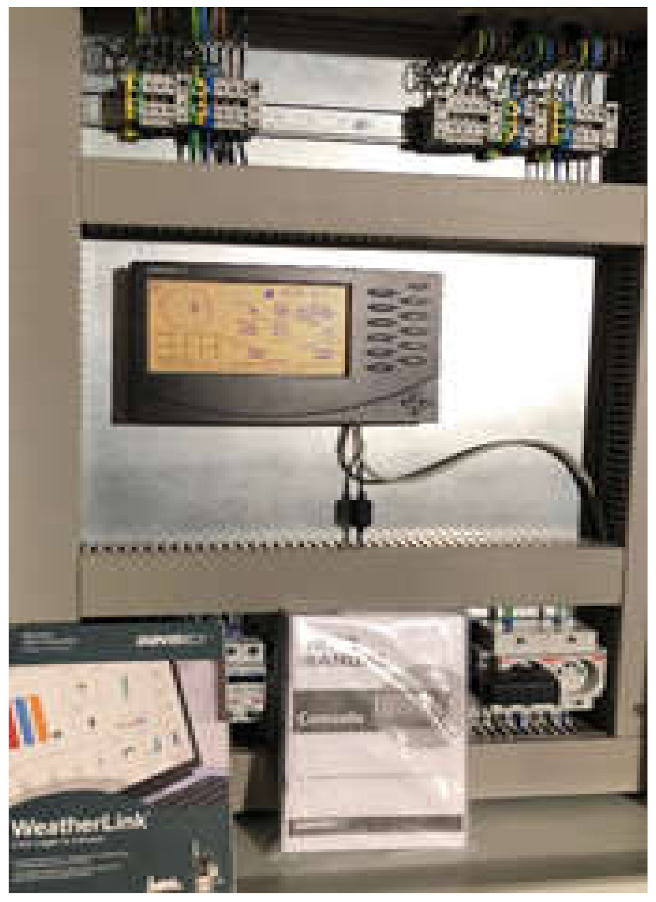
Installation monitoring and verifying the environmental parameters of the performance of a modernized ribbon-blade wind turbine. The installation verifying the environmental parameters of the modernized ribbon wind turbine is the end of the telecommunication sewage system monitoring (point 3—Figure 8). Source: the authors’ own materials (based on R. Kasner).

As a result of the research conducted, the following aims were achieved:

− Producing (Figure 7) and assembling a modernized ribbon-blade wind turbine at a research station (Figure 8, Figure 9 and Figure 10),
− Designing and obtaining administrative decisions for measurement-verification stations for wind turbines in target conditions (Figure 8),
− Constructing measurement-verification stations featuring an installation verifying environmental parameters and also featuring teletechnical canalization to verify the performance of various types of wind turbine constructions in target conditions (Figure 10 and Figure 11).

The range of the completed project raises the implementation readiness of a ribbon-blade wind turbine by having produced a modernized ribbon-blade wind turbine as well as research stations for said turbines featuring an installation for verifying the environmental parameters relating to their performance. The solution implemented makes it possible to undertake detailed testing of the prototype in target conditions (Table 9).

### 4.6. TRL 8: Producing the Final Version of the Product; TRL 9: Completion of a Test Series and Obtaining Product Conformity Certificates and Approvals for Use 

As part of the eighth level, multiple assessments of the innovative ribbon wind farm were carried out. As part of the final research, tests were carried out on the operational safety of the innovative ribbon wind turbine in the range of various wind speeds. The test results were verified positively. Producing the final version of the product and completing a test series and obtaining product conformity certificates and approvals for use.

A final, professional version of the wind turbine was produced. Talks and preparatory work are underway with professional wind turbine construction companies concerning the completion of a trial batch of wind turbines.

Evaluating the utility of the design idea of the working unit and ribbon blades analyzed herein with respect to the conversion of wind energy into useful energy—in other words, the evaluation of rotor performance—can be carried out by calculating the theoretical power of the system and relating it to the actual state. By actual state, we mean an operating wind turbine and the data to be compared to the theoretical calculations will be collected during said turbine’s operation.

**Figure 12:**
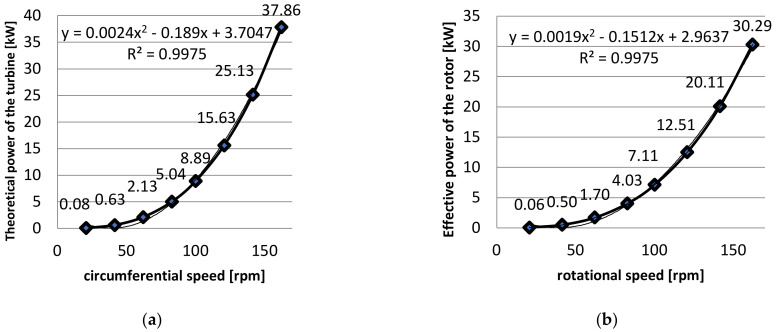
(**a**) Theoretical power as a function of speed; (**b**) Effective power as a function of speed. Source: the authors’ own materials.

In the section devoted to theoretical calculations, formulas (1)–(8) will be presented in the methodology. A variable wind speed VK in the range of (6 to 47) m·s^−1^ was assumed in the calculations. Table 10 and Table 11 show the calculations for the pressure and force distribution on the surface of the ribbons along with the theoretical powers, while Table 12 shows the forces and powers obtained, including efficiency. The most essential parameter speaking to the usefulness of the wind turbine is the power range provided by the system during its operation. Figure 12a presents theoretically generated powers as a function of rotor speed.

During the turbine’s operation, part of the power is used to overcome certain resistances resulting from the solutions applied. It was assumed that the efficiency of the turbine analyzed herein equals 0.8. By applying Equation (8) we derate the effective power, thereby obtaining a baseline for further calculations.

(8) $$\eta_{k}=\frac{N_{U}}{N_{T}}$$

where:

N_U_—effective power on the shaft of the turbine’s rotor, W,
N_T_—theoretical wind power acting on the ribbon-blade turbine, W.

(9) $$N_{U}=\eta_{k}\cdot N_{T}$$

Table 12 with Figure 12b shows the effective powers obtained on the ribbon-blade turbine.

The second part of the wind turbine analysis was the practical analysis offering the possibility to verify the results that were obtained from the theoretical part. The tests that were conducted relating to turbine measurements of actual practical power thus far yielded unsatisfactory results. On every occasion, weather-related conditions made it impossible to take proper measurements. Due to an insufficient amount of time, therefore, the decision was made to shift the practical analysis in time for different weather conditions, and to continue work. This approach made it possible to take proper measurements of the practical, effective power of the turbine (Figure 12b) and to undertake a more detailed theoretical analysis (Table 13).

## 5. Discussion

The objective of the study was achieved: to apply a methodology, conduct original research, and assess the technological readiness of a new ribbon-blade wind turbine in accordance with NASA’s TRL system. It was confirmed that to uphold an appropriate level of originality, it was necessary to complete an analysis of the current state of knowledge and technology in the field of innovation strategy and technological development and to assess the degree of technological readiness specific to the construction and operation of innovative machinery

On the basis of the tests conducted, it can be concluded that the structural form of the wind turbine unit analyzed herein featuring a new ribbon turbine design was technologically verified positively. These have a significant impact on the assessment indicators of the conversion process, these being efficiency, unit energy consumption, and the quality of the power and energy of the wind power plant. The total score of particular technological readiness levels is positive and high, with the exception of TRL level 9, which is related to obtaining product conformity certificates and approvals for use (Table 14).

The use of the new turbine design form in the windmill analyzed herein—a working ribbon unit—resulted in a technological increase in efficiency from 13% to 32% and a reduction in unit internal energy consumption from 18% to 36% compared to the traditional wind turbine design (as seen in Figure 10).

The NASA-based evaluation of the innovative wind turbine studied herein falls in line with the standards for smart development based on knowledge and innovation (EU 2020 Strategy). The empirical objective of the study was achieved through the use of original instrumentation and modern computer-aided procedures methodologically consistent with mechanics, construction and operation of machinery (CAE standards). The practical objective was achieved by conceiving and showing original design features and parameters relating to the innovation, investment, and research processes while satisfying the principles and rules of electric power engineering, construction and design theory, and above all, the expectations of manufacturers and operators regarding the possibility of manufacturing new wind turbines. Furthermore, the said objective was achieved by obtaining a high quality of the product (power and electricity), higher process efficiency (with respect to ecological, energetic and economic efficiency categories), and higher harmlessness in the impact of the product and process connected with the operation of an innovative ribbon-blade wind turbine, according to the idea of the scientific team from the University of Science and Technology in Bydgoszcz (Poland).

## Figures and Tables

**Table 1:** Readiness levels in accordance with the TRL method [1].

TRL Level	Characteristics
TRL 1	Identifying basic operating principles
TRL 2	Formulating technology concept
TRL 3	Proof-of-concept via experimentation
TRL 4	Validating the technology in laboratory conditions
TRL 5	Validating the technology in relevant environment
TRL 6	Prototype demonstration in simulated environment similar to the target one
TRL 7	Prototype demonstration in the target environment
TRL 8	Producing the final version of the product
TRL 9	Completing a test series and obtaining product conformity certificates and approvals for use

**Table 2:** Conclusion, assessment result of TRL level 1.

TRL 1	Identifying basic operating principles	Positive assessment of mechanical operating principles

**Table 3:** Using the PN-EN 1991–1-4:2008 norm, we can define the pressure of the wind speed with the following formula.

Dependence		Description
$q_{k}=\frac{\rho \cdot V_{k}^{2}}{2}\;\left[\mathrm{Pa}\right]$	(1)	where: q_k_—pressure of the wind speed $V_{k}^{2}$—wind speed in a given climate zone, ρ—air density 1.23 kg/m^3^.
After calculating the pressure of the wind speed we can calculate the characteristic load caused by wind effects with the following formula:		
$p_{k}=q_{k}\cdot \beta$ [Pa]	(2)	where: p_k_—characteristic load caused by wind effects, β—coefficient of wind gust action.
The design load applied when determining the ultimate limit states of the construction are derived from the formula:		
$p=p_{k}\cdot \gamma_{f}$ [Pa]	(3)	where: p_k_—characteristic load caused by wind effects, γf—load coefficient.
After determining the design load and surface area designated from the rotor’s ribbon we can compute the force applied to the relevant surface. The calculated force will be directed perpendicular to the surface from the calculations. This force can be derived from the following formula:		
$P_{w}=\frac{p}{A_{j}}$ [N]	(4)	where: P_w_—unit resultant force applied perpendicular to the surface, p—design load, A_j_—unit surface area of a part of the rotor’s ribbon [m^2^].
Knowing the resultant force on the unit area of the blade we can calculate the circumferential force needed to define the power of the turbine. To this end, we will use the following formula:		
$P_{\mathrm{OB}}=\cos\alpha \cdot P_{w}$ [N]	(5)	where: α—angle between force P_w_ and the frontal plane of the rotor P_w_—unit resultant force on the rotor.
The next step is to calculate the rotor power for different rotational speeds, which will be determined on the basis of wind speed variation. First, we derive the rotor speed from the formula:		
$n_{t}=\frac{60\cdot V_{n}\cdot \delta }{\pi \cdot D}$ [rpm]	(6)	where: V_n_—wind speed, δ—specific speed, D—rotor diameter.
Next we may proceed to calculate the theoretical power of the rotor:		
$N_{T}=\frac{\sum_{i}P_{\mathrm{OBi}}\cdot R\cdot n_{\mathrm{ti}}}{9550}$ [kW]	(7)	where: $\sum_{i}P_{\mathrm{OBi}}$—total circumferential forces occurring on particular planes of the rotor, R—active radius of the rotor, n_ti_—given rotational speed of the rotor.
Finally, when taking the assumed efficiency of the system into account, the power output will be calculated:		
$\eta_{k}=\frac{N_{U}}{N_{T}}$	(8)	where: $\eta_{k}$—efficiency of the system $N_{T}$—theoretical power which in practice is the wind power acting on the ribbon-blade turbine.

**Table 4:** Conclusion, assessment result of TRL level 2.

TRL 2	Formulating the solution concept	Positive assessment of the mechanical concept of the solution

**Table 5:** Conclusion, assessment result of TRL level 3.

TRL 3	Validating the proof-of-concept experimentally	Positive assessment, concept experimentally valid

**Table 6:** Material properties of steel AISI 304.

The Chemical Composition of Steel AISI 304								
Element	Iron	Chrome	Nickel	Manganese	Silicon	Coal	Potassium	Sulfur
Participation, %	66–74	18–20	8–10.5	Max 2	Max 1	0.08	0.045	0.03
Properties of steel AISI 304								
Properties			The value of the metric unit					
Density			7.9 × 10^3^				kg·m^−3^	
Modulus of elasticity			193				GPa	
Thermal expansion (20 °C)			17.2 × 10^−6^				°C ^−1^	
Specific heat capacity			502				J·(kg·K)^−1^	
Thermal conductivity			16.2				W· (m·K) ^−1^	
Electrical resistance			7.2 × 10^−7^				Ohm·m	
Tensile strength			520				MPa	
Yield point			210				MPa	
Elongation			45				%	
Hardness			<215				HB	
Melting temperature			1400–1450				°C	

**Table 7:** Conclusion, assessment result of TRL levels 4 and 5.

TRL 4	Validating the technology in laboratory conditions	Positive assessment, elements of technology verified in laboratory conditions
TRL 5	Validating the technology in simulated operational conditions	Positive assessment in laboratory conditions as simulated operational conditions

**Table 8:** Results of the assessment of environmental emissions of innovative wind farms.

Wind Power Plant	Global Warming (GWP100), kg CO_2_ eq	Renewable Wind Energy, MJ
Double Bladed	70.2	3.76
Three Bladed	77.8	4.7
Four bladed	93.3	5.64
Five Bladed	109	6.58
Six Bladed	124	7.52

**Table 9:** Conclusion, assessment result of TRL levels 6 and 7.

TRL 6	Production and demonstration of a prototype in a simulated environment similar to the target one	Assessment positive, prototype produced by professional wind turbine producer
TRL 7	Demonstration of the prototype in target operational conditions	Assessment positive, Prototype demonstrated in the target conditions of a laboratory

**Table 10:** Summary of wind speed pressure distribution and ribbon-blade surface area. Source: the authors’ own materials.

Lp.	Area A [m^2^]	Speed V_K_ [m·s^−1^]	Wind Speed Pressure q_k_ [Pa]	Characteristic Load p_k_ [Pa]	Design Load p [Pa]
1	0.105481	6	22	40	52
2	0.118495	12	89	159	207
3	0.105481	18	199	359	466
4	0.118495	24	354	638	829
5	0.105481	29 *	517	931	1210
6	0.118495	35	753	1356	1763
7	0.105481	41	1034	1861	2419
8	0.118495	47	1359	2445	3179

* marks the beginning of critical speeds and dangerous winds.

**Table 11:** Summary of the distribution of theoretical forces on the surface of the ribbon blades as well as the rotational speed and theoretical power on the rotor that were obtained. Source: the authors’ own materials.

Lp.	Speed V_K_ [m·s^−1^]	Resultant Force $\underset{i}{\sum }P_{wi}$ [N]	Circumferential Force $\underset{i}{\sum }P_{OBi}$ [N]	Revolutions nt [rpm]	Theoretical Power N_T_ [kW]
1	6	46	33	21	0.08
2	12	186	131	41	0.63
3	18	418	295	62	2.13
4	24	743	525	83	5.04
5	29 *	1084	767	100	8.89
6	35	1579	1117	121	15.63
7	41	2167	1533	141	25.13
8	47	2848	2014	162	37.86

* marks the beginning of critical speeds and dangerous winds.

**Table 12:** Summary of the distribution of the effective forces on the surface of the ribbon blades and the rotational speeds and effective power on the rotor. Source: the authors’ own materials.

Lp.	Speed V_K_·[m·s^−1^]	Circumferential Force $\underset{i}{\sum }P_{OBi}$ [N]	Revolutions nt [rpm]	Theoretical Power N_T_ [kW]
1	6	26	21	0.06
2	12	105	41	0.50
3	18	236	62	1.70
4	24	420	83	4.03
5	29 *	613	100	7.11
6	35	893	121	12.1
7	41	1226	141	20.1
8	47	1611	162	30.9

* marks the beginning of critical speeds and dangerous winds.

**Table 13:** Conclusion, assessment result of TRL levels 8 and 9.

TRL 8	Producing the final version of the product	Assessment conditionally positive, a final version of the product was completed
TRL 9	Completion of a test series and obtaining product conformity certificates and approvals for use	No assessment

**Table 14:** Levels and post-test readiness assessments in accordance with the TRL method.

TRL Level	Characteristics	Evaluation
TRL 1	Identifying basic operating principles	Positive, high
TRL 2	Formulating the solution concept	Positive, high
TRL 3	Validating the proof-of-concept experimentally	Positive, high
TRL 4	Validating the technology in laboratory conditions	Positive, high
TRL 5	Validating the technology in simulated operational conditions	Positive, high
TRL 6	Production and demonstration of a prototype in a simulated environment similar to the target one	Positive, high
TRL 7	Demonstration of the prototype in target operational conditions	Positive, high
TRL 8	Producing the final version of the product	Assessment conditionally positive, a final version of the product was completed
TRL 9	Completion of a test series and obtaining product conformity certificates and approvals for use	No assessment

## Data Availability

The data presented in this study are available on request from the corresponding author.
